# Heterologous fermentation of a diterpene from *Alternaria brassisicola*

**DOI:** 10.1080/21501203.2014.917735

**Published:** 2014-05-28

**Authors:** Julia Arens, Dominik Bergs, Mirja Mewes, Juliane Merz, Gerhard Schembecker, Frank Schulz

**Affiliations:** ^a^ Department for Chemistry and Biochemistry, Ruhr University Bochum, 44780Bochum, Germany; ^b^ Department of Biochemical and Chemical Engineering, TU Dortmund University, 44227Dortmund, Germany; ^c^ Department of Chemistry and Chemical Biology, TU Dortmund University, 44221Dortmund, Germany

**Keywords:** terpenes, heterologous fermentation, anti-tumour, metabolic engineering, directed evolution, enzyme engineering

## Abstract

A variety of different applications render terpenes and terpenoids attractive research targets. A promising but so far insufficiently explored family of terpenoids are the fusicoccanes that comprise a characteristic 5-8-5 fused tricyclic ring system. Besides herbicidal effects, these compounds also show apoptotic and anti-tumour effects on mammalian cells. The access to fusicoccanes from natural sources is scarce. Recently, we introduced a metabolically engineered *Saccharomyces cerevisiae* strain to enable the heterologous fermentation of the shared fusicoccane–diterpenoid precursor, fusicocca-2,10(14)-diene. Here, we show experiments towards the identification of bottlenecks in this process. The suppression of biosynthetic by-products via medium optimisation was found to be an important aspect. In addition, the fermentation process seems to be improved under oxygen limitation conditions. Under fed-batch conditions, the fermentation yield was reproducibly increased to approximately 20 mg/L. Furthermore, the impact of the properties of the terpene synthase on the fermentation yield is discussed, and the preliminary studies on the engineering of this key enzyme are presented.

## Introduction

**Figure 1.**
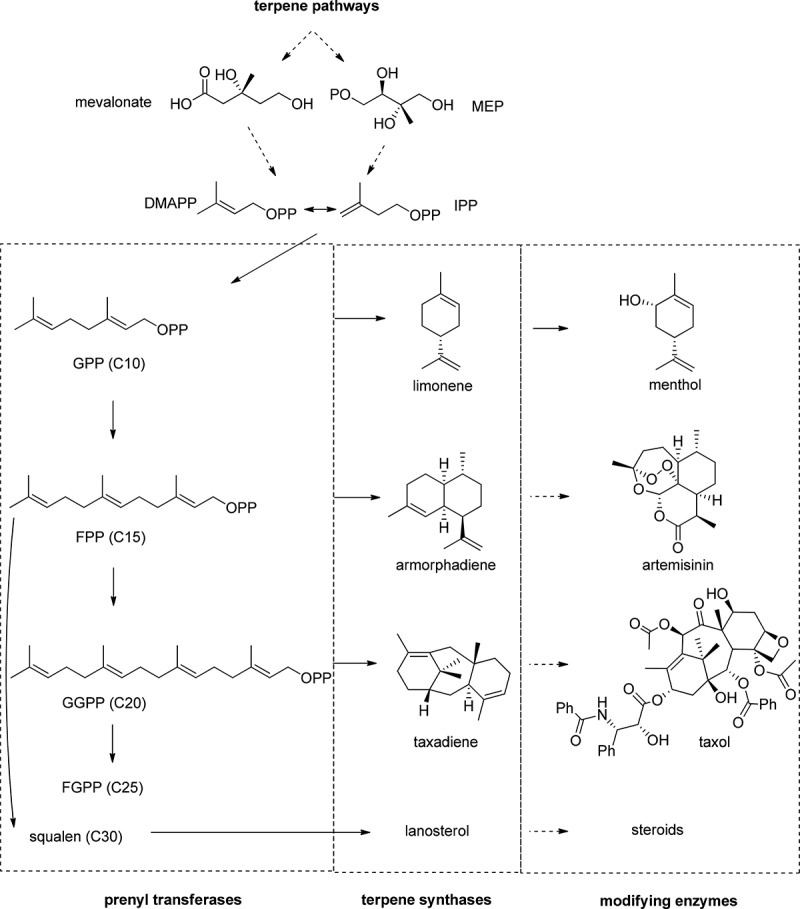
Schematic representation of terpene biosynthesis and structures of industrially relevant terpenoids like the flavour menthol, the anti-malaria compound artemisinin and the anti-cancer drug taxol. Dimethylallyl pyrophosphate (DMAPP) and isopentenyl pyrophosphate (IPP) are biosynthetically produced via the mevalonate and the 2-C-methyl-D-erythritol 4-phosphate (MEP) pathways. These terpene precursors are then converted by prenyl transferases to geranyl pyrophosphate (GPP), farnesyl pyrophosphate (FPP), geranylgeranyl pyrophosphate (GGPP), farnesylgeranyl pyrophosphate (FGPP) and bigger linear isoprenoids such as squalen. Terpene synthases are responsible for the conversion to terpene scaffolds. The insertion of functional groups is catalysed by a multitude of different enzymes to form bioactive terpenoids.

Fungi comprise a rich source of various natural products of pharmaceutical and other use. The first known antibiotic penicillin (Fleming 1929), the immunosuppressant cyclosporin (Dreyfuss et al. 1976) or the first isolated cholesterol-lowering 3-hydroxy-3-methylglutaryl-coenzyme A ( HMG-CoA) reductase inhibitor mevastatin (Endo et al. 1976) were primarily found to be produced in fungi. Besides non-ribosomal peptides and polyketides, fungi also produce several members of the largest class of natural products, the terpenes and typically oxyfunctionalised terpenoids. To date, over 50,000 structurally different terpenes and terpenoids are known (Conolly and Hill 1991). They play important roles as primary metabolites in several central anabolic and catabolic processes and are produced as secondary metabolites in all branches of life. Prominent pharmaceuticals like taxol^®^ as anti-cancer drug or artemisinin as an anti-malaria agent highlight the importance of terpenoids as research targets. All terpenoids are derived from the two universal isomeric C_5_ units – isopentenyl pyrophosphate (IPP) and dimethylallyl pyrophosphate (DMAPP) (Figure 1). Nature has evolved two distinctive pathways to synthesise these ubiquitous terpene building blocks. Eukaryotes and archaea typically use the mevalonate pathway (Bloch 1965), whereas the 2-C-methyl-D-erythritol 4-phosphate (MEP) pathway is found in most prokaryotes (Rohmer et al. 1993; Rohdich et al. 2002) and in the plastids of plants (Schwender et al. 1996). While prenyl transferases catalyse sequential chain elongations to linear prenyl pyrophosphates, terpene synthases catalyse further rearrangements or cyclisations to monoterpenes (C10), sesquiterpenes (C15), diterpenes (C20), sesterterpenes (C25) or bigger terpenes (Figure 1). While only a handful of the linear prenyl pyrophosphates are produced during biosynthesis, a number of different terpenes are formed through different cyclisation mechanisms, triggered by differently shaped active sites of the native terpene synthases (Christianson 2006). Often, terpenes are furnished with diverse functionalisations, most prominently hydroxylations, to enable specific interactions with their biological targets.

A ubiquitous and yet underexplored family of terpenes are the fusicoccanes. These compounds are found as diterpenoids and sesterterpenoids in fungi, algae, higher plants and insects and are characterised by their 5-8-5 tricyclic structural motif (Muromtsev et al. 1994).

**Figure 2.**
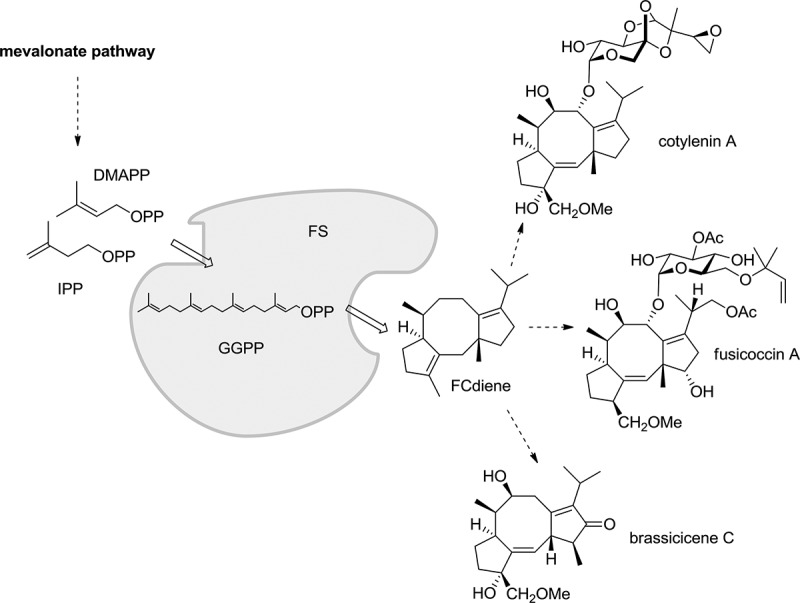
Biosynthesis of fusicocca-2,10(14)-diene (FCdiene). The bimodular FCdiene synthase (FS) catalyses both the reactions for the formation of GGPP and the cyclisation to FCdiene (Toyomasu et al. 2007). During a number of successive enzymatic steps, fusicoccanes like cotylenin A, fusiccocin A or brassicicene C are thought to be produced from this central precursor.

A fusicoccane subfamily comprising diterpenoids like fusicoccins (Ballio et al. 1964), cotylenins (Sassa et al. 1970) and brassicicenes (MacKinnon et al. 1999) is preferentially produced by various phytopathogenic fungi. Such diterpenoids are identical in their carbon skeleton and stereochemical configuration but vary in their substitution and degree of functionalisation. Until now, two enzyme isoforms from distinct fusicoccane-producing fungi were isolated and shown to catalyse identical reactions (Toyomasu et al. 2007; Minami et al. 2009) towards the shared precursor (+)-fusicocca-2,10(14)-diene (FCdiene) (Figure 2). Both FCdiene synthase (FS) isoforms are bimodular and consist of a prenyl transferase domain and a terpene cyclase domain. These enable the synthesis of the diterpene FCdiene (C_20_) directly from the two universal isoprenoid C_5_ building blocks IPP and DMAPP (Toyomasu et al. 2007). The bioactivity of this subfamily of fusicoccanes results from the stabilisation of protein–protein interactions between eukaryotic 14-3-3 proteins and their partner proteins (Würtele et al. 2003; Yang et al. 2006). Fusicoccin A, for example, permanently stabilises the 14-3-3/plant H^+^–ATPase interaction (Würtele et al. 2003), thereby causing wilting of the treated plant (Ballio et al. 1964). The crystal structure of a ternary complex between cotylenin A, a plant 14-3-3 isoform and an H^+^–ATPase phosphopeptide was determined (Ottmann et al. 2009), showing an apparently similar activity of cotylenin A. Besides herbicidal activities, the fusicoccanes also have effects on amphibian embryogenesis (Bunney et al. 2003) and on human cancer cells. Fusicoccin A and cotylenin A were found to induce apoptosis in tumour cells in combination with the immunosuppressant Interferon-α (Honma et al. 2003a, 2003b, 2005; de Vries-van Leeuwen et al. 2010). But in contrast to fusicoccin A, cotylenin A was also found to induce differentiation of myeloid leukaemia cells (Asahi et al. 1997; Matsunawa et al. 2006). Because of a different oxidation pattern, cotylenin A can bind to binary complexes between 14-3-3 proteins and so-called mode I or mode II phosphorylated binding motif peptides (Ottmann et al. 2009), which can be found in mammalian proteins like Raf kinases, Cdc25 phosphatases or the transcription factor Miz1 (Muslin et al. 1996; Yaffe et al. 1997). The potential of cotylenin A as anti-cancer compound was further verified by its activity in xenograft mouse models (Honma et al. 2003a, 2003b; Kasukabe et al. 2005). However, access to cotylenin A has dwindled because of the inability to re-cultivate the producing fungus *Cladosporium* sp. 507-1W under laboratory conditions due to loss of proliferation (Minami et al. 2009; Ono et al. 2011). Chemical routes to complex diterpenoids and terpenes like (−)-cotylenol (Kato et al. 1996), (+)-taxadiene (Mendoza et al. 2011) and FCdiene (Kato et al. 1998) are reported but are rare and not yet generalised or only manageable on an analytical scale. Only recently, it became possible to biosynthesise preparative amounts of important terpenoids by means of heterologous fermentation in model organisms. Key to this endeavour is extensive metabolic engineering of the host strain. A famous, but also unique, example is the biosynthetic access of artemisinic acid and subsequent synthetic transformation to artemisinin (Paddon et al. 2013). The field, however, is characterised by a lack of generalisable strategies to access complex terpenes and terpenoids in preparative amounts.

Natural products are often considered to show higher structural complexity than required for human application (Wach and Gademann 2012). Often, reduction of high-molecular-weight natural products to simplified molecules targeting the same effector proteins increases their bioavailability. This strategy may also work for fusicoccanes. Previous studies suggest that parts of fusicoccin A are not required for target binding, in particular the complex carbohydrate decoration (Ballio et al. 1981; Würtele et al. 2003).

To enable access to fusicoccane-type molecules, we aimed for a strategy that was based on the heterologous production and a subsequent enzymatic or synthetic elaboration of the 5-8-5 tricyclic fusicoccane precursor FCdiene. Therefore, in initial studies, we investigated three potential fermentation strains for the microbial production of FCdiene (Arens et al. 2013). To find a strain that is capable to produce preparative amounts of FCdiene, the expression of the bimodular FS from the filamentous fungus *Alternaria brassicicola* (AbFS) (Minami et al. 2009), was successfully achieved in the bacterium *Escherichia coli*, the filamentous fungus *Aspergillus nidulans* and the yeast *Saccharomyces cerevisiae. S. cerevisiae* was able to produce the highest amounts of the desired diterpene. Subsequently, a method was developed to isolate FCdiene in high purity and multi-milligram scale, which paves the way both for further optimising the system and accomplishing high production levels of fusicoccane derivatives.

We here describe a characterisation of the heterologous fermentation of FCdiene in depth with regard to biological and biosynthetic aspects. In particular, we discuss potential bottlenecks in the fermentation and give first hints towards its improvement.

## Materials and methods

### Fermentation of FCdiene by *S. cerevisiae*

For fermentation of FCdiene, *S. cerevisiae* CEN.PK2-1c [pRS313-*upc2.1*, pRS315-*thmgr*, pVV214-*abfs*] was applied (Arens et al. 2013). Pre-cultures were grown in synthetic dropout (SD) medium under selection for auxotrophic markers (SD-His-Leu-Ura: 0.67% w/v yeast nitrogen base without amino acids, 0.001% w/v adenine, 0.005% w/v Arg HCl, 0.008% w/v Asp, 0.005% w/v Ile, 0.005% w/v Lys, 0.002% w/v Met, 0.005% w/v Phe, 0.01% w/v Thr, 0.002% w/v Trp, 0.005% w/v Tyr, 0.014% w/v Val, 2% w/v glucose) at 30°C to an OD_600_ of 1. After centrifugation, cells were diluted into 10 volumes of either YPD (1% w/v yeast extract, 2% w/v peptone, 2% w/v glucose) (Engels et al. 2008; Arens et al. 2013) or SD-His-Leu-Ura. All fermentation media were supplemented with 1 mM MgCl_2_ (Carl Roth). Shaking flask cultures were grown in a medium- to-flask volume ratio of 1:5 in an orbital shaker (Multitron Standard, Infors HAT with 5-cm displacement) for 48 h at 30°C and 110 rpm or at 180 rpm for increased aeration.

Fed-batch cultivations were run in 500 mL SD-His-Leu-Ura supplemented with 1 mM MgCl_2_ and 20 mM succinate (Carl Roth) in a 2-L glass stirring tank incubator without pH control at 30°C for 5 days. Stirring frequency was held constant at 600 rpm (IKA RCT classic stirrer) and up to 9.2 L/min air were supplied (Sera^®^ precision air 550r plus). The batch culture with 2% (w/v) glucose as initial sugar concentration was fed once with 1.5% (w/v) glucose after 21–22 h in the exponential growth phase.

### Quantification of FCdiene

After extraction of 2 mL of culture with *n*-pentane (HPLC-grade, Sigma-Aldrich), the extract was analysed by gas chromatograph with flame ionization detector using cycloundecane (Sigma-Aldrich) as internal standard. Importantly, in these experiments, the hydrophobic adsorption resin reported to enhance the FCdiene yield (Arens et al. 2013) was omitted as it would have blurred the quantification results. Quantifications were performed in triplicate.

### GC measurements

gas chromatograph-mass spectrometer coupling (GC/MS) measurements were performed on an Agilent Technologies 7890A GC System with a flame ionisation detector, a 5975C inert XL MSD Triple-Axis Mass Detector and Agilent 19,091S-433 Trace Analysis column. GC-conditions: 1 µL of sample was injected with an evaporation temperature of 250°C, 1.8 bar, 2.5 mL/min, split 20:1. He carrier gas, temperature gradient 50°C/1 min, 40°C/min gradient 300°C, 300°C/5 min.

### Construction of a *S. cerevisiae* fermentation strain harbouring a codon optimised version of abfs

The codon optimised version of *abfs* for *S. cerevisiae* (*abfsSC*) was ordered from Eurofins MWG. The optimised gene was inserted into the yeast expression vector pVV214 by the Gateway^®^ cloning technology using the pDONR221 vector for the construction of pEntry clones. Therefore, the gene-specific forward 5’-GGG GAC AAG TTT GTA CAA AAA AGC AGG CTT CAA AAA TGA AGT ACC AGT TCT CCA TCA TTG-3’ and reversed 5’-GGG GAC CAC TTT GTA CAA GAA AGC TGG GTT TAC AAT TTC AAC ATC ATC AAC ATT AAT TC-3’ primers were applied. Correct constructs were isolated by alkaline lysis and ethanol precipitation (Birnbiom and Doly 1979) from chemically competent *E. coli* OmniMAX^TM^ 2 T1^R^ cells (Invitrogen) for verification by sequencing (StarSEQ GmbH). The yeast mutant harbouring pRS313-*upc2.1* and pRS315-*thmgr* was co-transformed with pVV214-*abfs* by the lithium acetate method (Amberg et al. 2005).

### Directed evolution of abfs

#### Design of a screening construct

To screen for soluble AbFS variants via blue-white screening on X-gal-containing agar plates (Wigley et al. 2001), a construct encoding for AbFS in C-terminal fusion to lacZα over a flexible linker was designed. The construct was based on the pBADM backbone series to enable an adjustable expression dependent on the arabinose concentration. This screening construct was prepared by the assemblage of three PCR amplicons by SLIC-MIX (Kushnir et al. 2012; Sundermann et al. 2013). Amplicon 1 was amplified from pBADM-11 (EMBL Heidelberg) with the forward 5ʹ-TGA TAA *GGC GCC* AGC TTG GCT GTT TTG GCG GAT G-3’ and reversed 5’-GGT ATT TCA TAG *CCA TGG* TTA ATT CCT CCT GTT AGC CCA AAA AAC G-3’ oligonucleotides. This way the His_6_-tag and the multiple cloning site of the vector were removed and two unique *Kas*I and *Nco*I sites (underlined sequences) were introduced into the screening construct backbone. Amplicon 2 consists of *abfs* without its stop codon but with restriction sites for *Nco*I upstream of *abfs* and *Not*I fused to a coding sequence for a linker fragment (same amino acid sequence as used by Reetz and Zheng 2011) downstream of *abfs*. These sites were introduced using the oligonucleotides 5’-AGG AGG AAT TAA *CCA TGG* CTA TGA AAT ACC AAT TTT CCA TCA TGG TGG-3’ and 5’-CAG CAG ATC CAG CAG ATC CT*G CGG CCG C*AA GCT TGA GCA TCA TTA GCA TCA G-3’. The generated restriction sites served for an easy insertion of *abfs* mutants by restriction and ligation after *ep*PCR. Amplicon 3 was amplified from pUC18 with the oligonucleotides 5’-GGA TCT GCT GGA TCT GCT GCT GGT TCT GGC GCA TCT ATG ACC ATG ATT ACG AAT TCG AGC-3’ and 5’-TCC GCC AAA ACA GCC AAG CT*G GCG CC*T TAT CAG CGC CAT TCG CCA TTC AG-3’ to yield *lacZα*. This enabled the introduction of a coding sequence for a linker region upstream of *lacZα* and a restriction site for *Kas*I downstream of the amplified gene. Wild-type *abfs* encoding construct was used as negative control, and pBADM-11-*lacZα* served as positive control.

#### *ep*PCR experiments and screening of soluble enzyme variants

For random mutagenesis by error-prone PCR, *Taq* polymerase (NEB) in standard Taq buffer (NEB) was used. Final concentrations of 7 mM MgCl_2_, 0.2 mM each of deoxyguanosine triphosphate and deoxyadenosine triphosphate, 1 mM each of deoxycytidine triphosphate and deoxythymidine triphosphate and varied concentration of MnCl_2_ (0–0.05 mM) were applied to reveal different mutation rates (Cirino et al. 2004). Primers previously used for amplicon 2 during design of the screening construct were used. PCR profile: 35 cycles, 1. 95°C/30 s 2. 50°C/30 s (initial 5 cycles)/62°C/30 s (final 30 cycles) 3. 68°C/2 min 4. 68°C/5 min final extension. Digested PCR products (*Dpn*I, *Nco*I and *Not*I from NEB) were extracted with Roti^®^-Phenol/Chloroform/Isoamyl alcohol (Carl Roth), precipitated with ethanol and ligated into the *Nco*I and *Not*I (NEB) digested screening vector backbone. Chemically competent *E. coli* OmniMAX^TM^ 2 T1^R^ (Invitrogen) were transformed with the ligation product and plated on LB agar plates (1% w/v tryptone, 0.5% w/v yeast extract, 1% w/v NaCl, 1.5% agar pH 7.4) containing 100 µg/mL carbenicillin, 40 µg/mL X-gal and 10 mM arabinose. After incubation at room temperature for 2.5 days, blue colonies were verified by restriction analysis and sequencing (StarSEQ GmbH). After expression in liquid medium, solubility and functional activity of obtained mutants were determined by sodium dodecyl sulfate polyacrylamide gel electrophoresis (SDS-PAGE) (Laemmli 1970) analysis and GC/MS measurements.

### Splitting and expression of the two domains of AbFS in *E. coli*

The two catalytic domains FCdiene cyclase (FCyc) and geranylgeranyl pyrophosphate synthase (GGPPS) of the bimodular fusicoccadiene synthase (Toyomasu et al. 2007) were amplified from *abfs* using the following oligonucleotides: *fcyc*-fw 5’-TTA TTT TCA GGG CG*C CAT G*GC AAA ATA CCA ATT TTC CAT CAT TGT GG-3’, *fcyc*-re 5’-AAG CTC TCG AGT *GCG GCC GC*T TAT CAC TGG TTG AAA CGC TTC TCA G-3’, *ggpps*-fw 5’-TTA TTT TCA GGG CG*C CAT G*GC AAC TCA ACT AGA TTG GAT GCA AAA TG-3’ and *ggpps*-re 5’-AAG CTC TCG AGT *GCG GCC GC*T TAT CAA AGC TTG AGC ATC-3’. Cloning of each segment into the backbone of pETM-11 (EMBL Heidelberg) was achieved via restriction and ligation cloning using *Nco*I and *Not*I (italicized). After verification of both constructs by sequencing (StarSEQ GmbH), *fcyc* and *ggpps* were expressed separately in *E. coli* BL21-Gold(DE3) (Agilent Technologies). After induction of expression at OD_600_ 1–1.3 with 1 mM Isopropyl *β*-D-1-thiogalactopyranoside (AppliChem), cells were cultivated at 19°C for 2 days in *terrific broth* medium (1.2% w/v tryptone, 2.4% w/v yeast extract, 0.4% v/v glycerine, 17 mM KH_2_PO_4_ and 72 mM K_2_HPO_4_) supplemented with 1 mM MgCl_2_.

## Results and discussion

### Heterologous fermentation in *S. cerevisiae*

**Figure 3.**
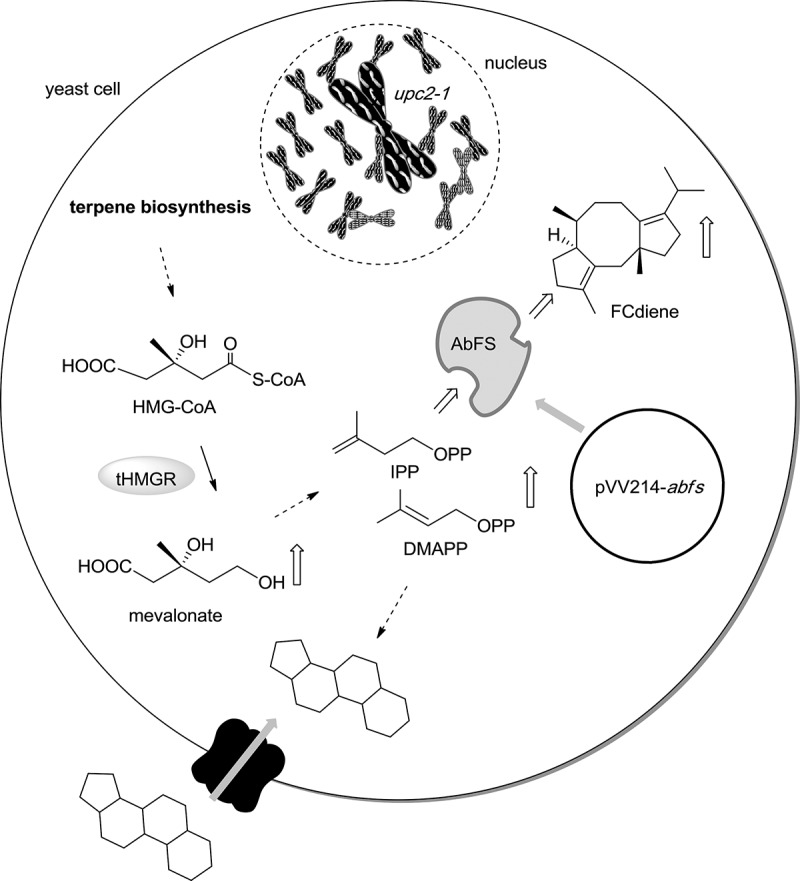
Schematic representation of the *S. cerevisiae*-mutant engineered for the production of FCdiene. Expression of *thmgr1* (gene encoding for a truncated version of the HMG-CoA (hydroxy-methyl-glutraryl coenzyme A) reductase isoenzyme 1) enabled an increased supply of isoprenoid precursor molecules due to deletion of its N-terminal regulatory domain. Mutation of the gene encoding for the transcription factor UPC2 influences the steroid metabolism and was used to further increase the flux towards heterologous produced terpenes. The expression of the recombinant FCdiene synthase was ensured by the co-transformation of the engineered yeast with the vector pVV214-*abfs.*

In recent years, *S. cerevisiae* has been introduced for the heterologous production of various terpenes and terpenoids. Intensive optimisation studies enabled, in several cases, its preparative use (reviewed by Misawa 2011). Often, however, the yield of the targeted terpenoids is unsatisfying. Recently, we constructed a mutant of *S. cerevisiae* CEN.PK2-1c (van Dijken et al. 2000; Engels et al. 2008) for the preparative fermentation of the diterpene FCdiene (Figure 3) (Arens et al. 2013). This strain was transformed with pVV214-*abfs*, which enabled the expression of the fungal FS isolated from cabbage – pathogen *Alternaria brassicicola* UAMH 7474 (AbFS) in *S. cerevisiae* under the control of the strong and constitutive phosphoglycerate kinase 1 promoter (Van Mullem et al. 2003). To increase the terpene levels, this strain was furthermore modified with a plasmid-born truncated version of hydroxy-methyl-glutraryl coenzyme A reductase isoenzyme 1 (tHMGR1) (Donald et al. 1997). In its wild-type form, this enzyme is strongly regulated to control terpene levels and catalyses the main rate-limiting step in the mevalonic acid pathway. Removal of its N-terminal regulatory domain delivers a regulation-insensitive variant. This leads to the accumulation of pathway intermediates under aerobic and semi-anaerobic conditions and can thereby lead to increased levels of heterologously produced terpenes in *E. coli* (Martin et al. 2003) as well as in *S. cerevisiae* (Jackson et al. 2003; Ro et al. 2006). Furthermore, the heterologous fermentation strain carried a plasmid that encoded for a mutated version of the transcription factor UPC2 (UPC2-1) (Lewis et al. 1988; Crowley et al. 1998). UPC2 is important for the regulation of the ergosterol content in yeast (Crowley et al. 1998; Vik and Rine 2001). Whereas import of sterols occurs in wild-type *S. cerevisiae* under anaerobic conditions (Trocha and Sprinson 1976), when the oxygen-dependent biosynthesis of sterols is suppressed, a single point mutation in the C-terminal region of the UPC2 transcription factor enables the uptake of sterols from the nutrient medium under aerobic fermentation conditions (Lewis et al. 1988; Crowley et al. 1998). This mutation was introduced into the FCdiene-producing strain to also yield improved FCdiene titres. In shaking flask fermentations, the use of this strain yielded 6 mg/L FCdiene in an isolated form (Arens et al. 2013).

Upon a more detailed analysis of the fermentation by mass spectrometry, we now found a substantial accumulation of the shared steroid precursor squalene in this yeast culture. This indicates that the metabolic flux towards triterpenes exceeds the demand, presumably by a reduced need for steroids and a concomitantly increased C_5_-precursor level (induced both by the tHMGR1 and the UPC2-1 mutations). It was previously shown that a downregulation of squalene synthase activity could further increase product titres for amorphadiene production (Ro et al. 2006).

**Figure 4.**
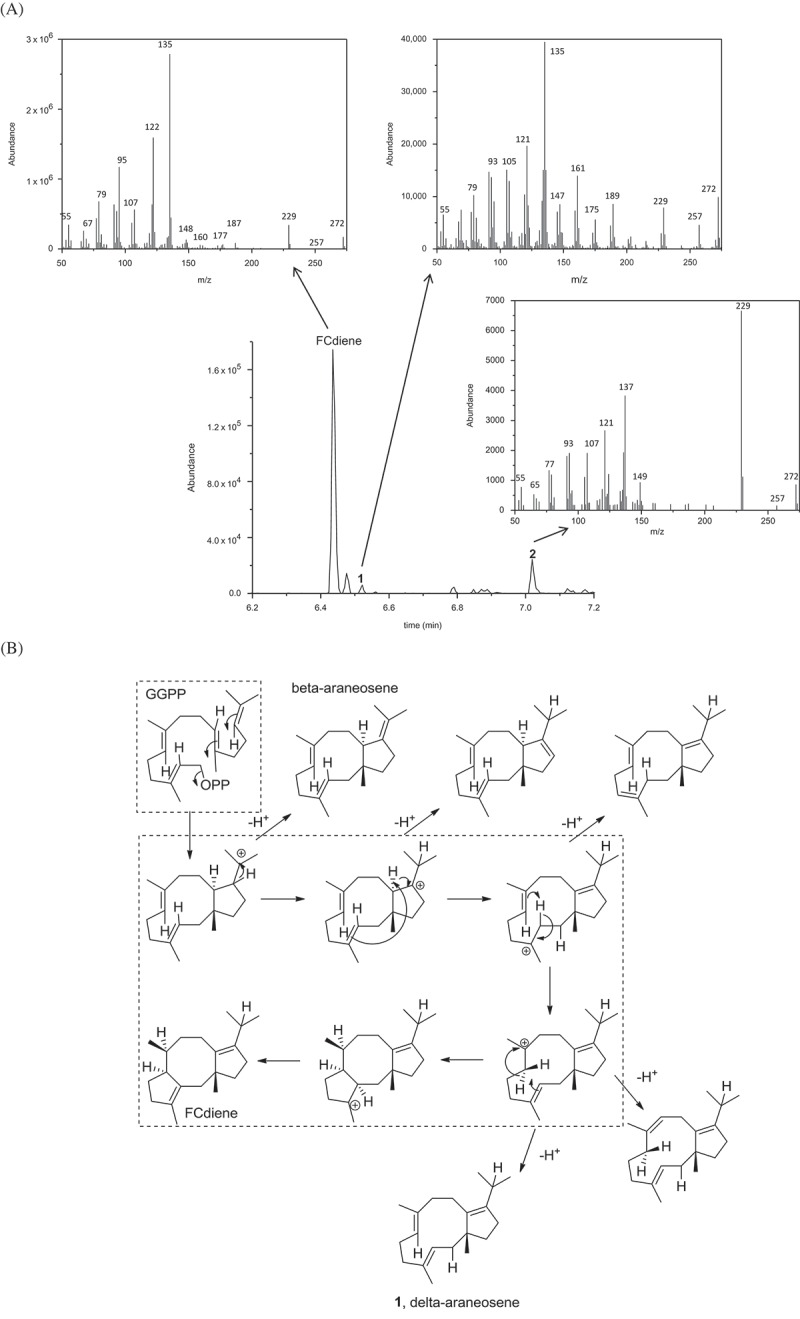
(A) Compounds **1** and **2** were found to be produced by the FCdiene fermentation strain with a similar GC mass fragmentation pattern to FCdiene and an M^+^ peak of 272. (B) Proposed cyclisation of FCdiene (Toyomasu et al. 2007) and potential shunt products. The proposed mechanism for the cyclisation to FCdiene is highlighted in the dashed boxes.

Now, experiments revealed that the fermentation of the previously reported yeast strain in YPD medium yields not only FCdiene but also two specific by-products (Figure 4(A), compounds **1** and **2**). Gas chromatography-electron ionization mass spectrometry analysis revealed these as intermediates or shunt products of FCdiene biosynthesis (Figure 4). Compound **1** has a similar fragmentation pattern to *δ*-araneosene (Jenny and Borschberg 1995), which was shown to be produced *in vivo* by the fusicoccin-producing fungus *Phomopsis amygdali* (Sassa et al. 2004) as well as *in vitro* by the purified FS from *P. amygdali* (PaFS) itself (Toyomasu et al. 2007). It is a neutral intermediate of the GGPP cyclisation towards FCdiene (Sassa et al. 2004; Tantillo 2011). To our knowledge, compound **2** has not been isolated before; its fragmentation pattern and absolute mass, however, strongly suggest it to be a double-bond isomer of compound **1** and as such rather a shunt product than an intermediate. These findings indicate that the activity of AbFS was too low to ensure rapid turnover of potentially accumulating intermediates.

These new findings on the fermentation of FCdiene by the recombinant yeast strain motivated us to examine the FCdiene fermentation in more detail.

#### Optimisation of fermentation conditions

To begin, different fermentation parameters were varied systematically in shaking flasks.

##### pH effects on FCdiene batch fermentations

**Figure 5.**
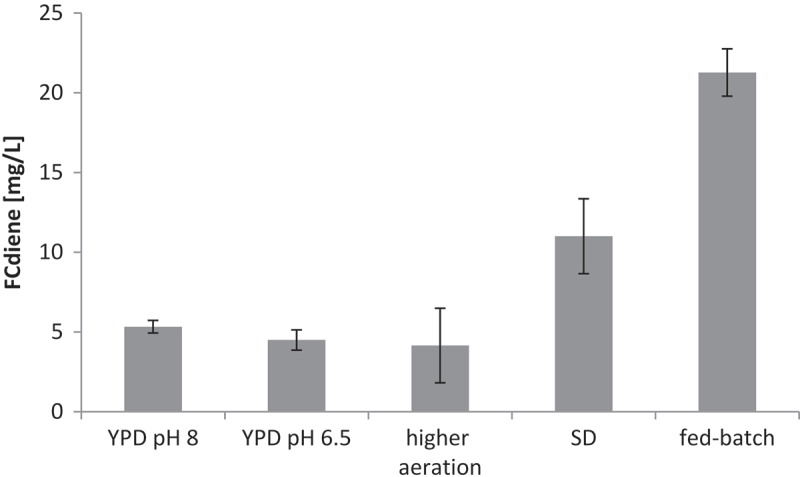
Comparison of FCdiene yields achieved under different fermentation conditions.

The effect of the external pH during fermentation was studied. To prevent isomerisation of the acid-sensitive FCdiene, we buffered the fermentation medium initially with 100 mM Tris-HCl to pH 8 (Engels et al. 2008; Arens et al. 2013). During fermentation, pH dropped to 6.5 during the first 14 h, later on it reached pH 8 again. These pH shifts can be explained by the metabolic conversion of glucose to short organic acids in the beginning of the fermentation, whereas in the end of the fermentation, presumably amino acids are degraded to alkaline ammonium ions. The Tris buffer was apparently too weak to override this effect. We examined crude extracts after fermentation in Tris-buffered YPD (with initial pH 8) and in unbuffered YPD medium (with initial pH 6.5). The fermentation was found to be not affected by the pH of the medium under the tested conditions (Figure 5).

##### Correlation between oxygen levels and FCdiene fermentation

We initially assumed that biomass production correlates with FCdiene yields. To study this hypothesis, we increased the oxygen transfer rate into the fermentation cultures by raising the shaking frequency. Indeed, increased biomass was observed in these cultures (OD_600_ ~ 30) compared to cultures grown with lower oxygen (OD_600_ ~ 20), but production of FCdiene was found to be unreliable in those fermentations. On the average, a lowered productivity of the fermentation was observed (see Figure 5). Although this effect was found to be statistically insignificant on laboratory scale, it was reproduced on a preparative scale with hydrophobic adsorbant added for *in situ* product removal. High levels of oxygen hence seem to have a negative effect on the fermentation of FCdiene.

##### Variation of fermentation media and fermentation processing

A common issue in heterologous fermentation, and in fact many heterologous protein expression experiments, is the genetic instability of the genetically modified organisms (Studier 2005). As described earlier, the FCdiene-producing yeast strain carries three different recombinant plasmids. Fermentation in the complex medium YPD did not apply selection pressure on the maintenance of these constructs. Now, control experiments showed that under those fermentation conditions, the strain loses all three plasmids within 20 h of cultivation. Based on these results, we tested the production of FCdiene in the selective medium SD without histidine, leucine and uracil (SD-His-Leu-Ura), thereby selecting for the maintenance of the three auxotrophic markers. Although biomass levels were lower compared to fermentations in YPD (OD_600_ ~ 13), the switch to the selective medium increased FCdiene levels twofold (Figure 5), presumably as a result of higher genetic stability of the fermentation strain. To further increase the FCdiene yield, the batch culture was fed once with 1.5% glucose during exponential growth in a 2-L stirring tank without pH control. To prevent the strong acidification to pH 2.5 of the SD-His-Leu-Ura medium during fermentation, starting from pH 5, succinate was added as metabolic buffer (Cha et al. 1998; Studier 2005). As an intermediate in the TCA cycle, besides its effect as buffer, succinate can be metabolised as an additional carbon source in yeast. When added from the beginning, succinate could stabilise the pH to 4.5 to 5 during long periods of fermentation, and the buffering effect was thus much stronger than in case of Tris-HCl. Through these improvements, the yield of FCdiene could be increased to approximately 20 mg/L (Figure 5). Importantly, fermentation in SD medium diminished the production of the FCdiene isomers **1** and **2** and thus rendered the fermentation not only more productive but also more specific. This suggests an increased activity of AbFS under the altered conditions, indicating that the enzyme itself might be the limiting factor.

A basic aspect in the preparative fermentation of hydrophobic terpenes is the prevention of product inhibition due to toxic effects on the host (Brennan et al. 2012) and the reduction of evaporation of volatile terpenes (Newman et al. 2006). This is typically achieved by the addition of a second hydrophobic phase to the fermentation medium to trap the desired metabolite quantitatively. The frequently used additive dodecane (Newman et al. 2006) was found to have a similar boiling point to FCdiene and was therefore difficult to separate. Since shorter alkanes with a lower boiling point had toxic effects on the fermentation host, we used in our initial studies the expensive solid C18 silica gel (Engels et al. 2008) for *in situ* product adsorption. As a cheaper alternative, we found that the polystyrene Lewatit^®^ VP OC 1064 MD PH with similar adsorption properties towards FCdiene enabled a simpler workup to isolate the desired fermentation product in preparative amounts. By supplying the polymeric adsorber resin at 6 g/L to the fermentation broth, the FCdiene yield was improved in the same manner as the previous used C18 silica gel (Arens et al. 2013) compared to fermentations without such an additive.

#### Exploration of further potential bottlenecks in the fermentation

Small but significant improvements of the FCdiene titres can be induced by systematic variation of fermentation parameters. Achieving an enhancement by several orders of magnitude, however, would be extremely time-consuming by these means as seen in the hallmark example artemisinin and its biosynthetic intermediates (Ro et al. 2006, 2008; Shiba et al. 2007; Dietrich et al. 2009; Westfall et al. 2012; Paddon et al. 2013).

While the directed *metabolic engineering* of heterologous hosts for the production of isoprenoids often has a significant impact on the yield, the product titre of individual metabolites observed varied strongly (Table 1). These findings suggest that besides the improvement of the C_5_ precursor supply, other inherent limitations might have to be addressed.

**Table 1.** Comparison of FCdiene yield in yeast with the heterologous production of other important terpenes and terpenoids.

Terpene	Used cell type	Improvement	Yield	Reference
FCdiene	*S. cerevisiae*	Metabolic engineered	20 mg/L in 120 h	This study
Taxadiene	*S. cerevisiae*	Metabolic engineered	8.7 mg/L in 48 h	Engels et al. (2008)
Taxol^®^	*Taxus baccata* cell cultures	Immobilised cells	43 mg/L in 16 days	Bentebibel et al. (2005)
Amorphadiene	*S. cerevisiae*	Metabolic engineered	41 g/L in 116 h	Westfall et al. (2012)
Artemisinic acid*	*S. cerevisiae*	Metabolic engineered	25 g/L in 160 h	Paddon et al. (2013)
Other sesquiterpenes	*S. cerevisae*	Metabolic engineered	0.37–40 mg/L	Jackson et al. (2003), Asadollahi et al. (2008), Asadollahi et al. (2009), Albertsen et al. (2011), Nguyen et al. (2012)
Limonene	*E. coli*	Metabolic engineered, artificial mevalonate pathway	430 mg/L in 72 h	Alonso-Gutierrez et al. (2013)

Note: *: with subsequent conversion to artemisinin by semi-synthesis.

Attempts to overcome potential limitations due to rare tRNAs in yeast by introduction of a codon optimised version of *abfs* for *S. cerevisiae* revealed no further improvement of FCdiene production. Hence, we concluded that the expression rate of *abfs* in yeast is not limiting.

Rather, the inherent catalytic ability or stability of the enzyme could be crucial to improve heterologous production of the desired compound (Leonard et al. 2010; Lauchli et al. 2013). Since there is no structural information for AbFS or closely related enzymes available that would allow for any rational mutagenesis approach to this challenge, we opted for *directed evolution* to improve its soluble expression. Random mutagenesis of the full-length terpene synthase gene via *error-prone PCR* was performed at different mutation rates (1–5 mutations/kb). The libraries of mutated genes were expressed in *E. coli*. This implies that folding stability and solubility are intrinsic and related properties of the enzyme and independent from the host, provided that the enzyme is correctly folded after translation. The lack of structural information and the size of AbFS (84 kDa) required an agar-plate-based screening for soluble expression. Therefore, *abfs* was C terminally fused to the lacZα-fragment of β-galactosidase. Soluble expression of the fusion protein should enable X-gal staining of an expression host carrying the *lacZΔM15* mutation (Wigley et al. 2001; Reetz and Zheng 2011). More than 30,000 clones were screened by this β-galactosidase complementation assay. However, no AbFS variants with a reproducibly higher soluble expression than the wild-type enzyme were obtained.

**Figure 6.**
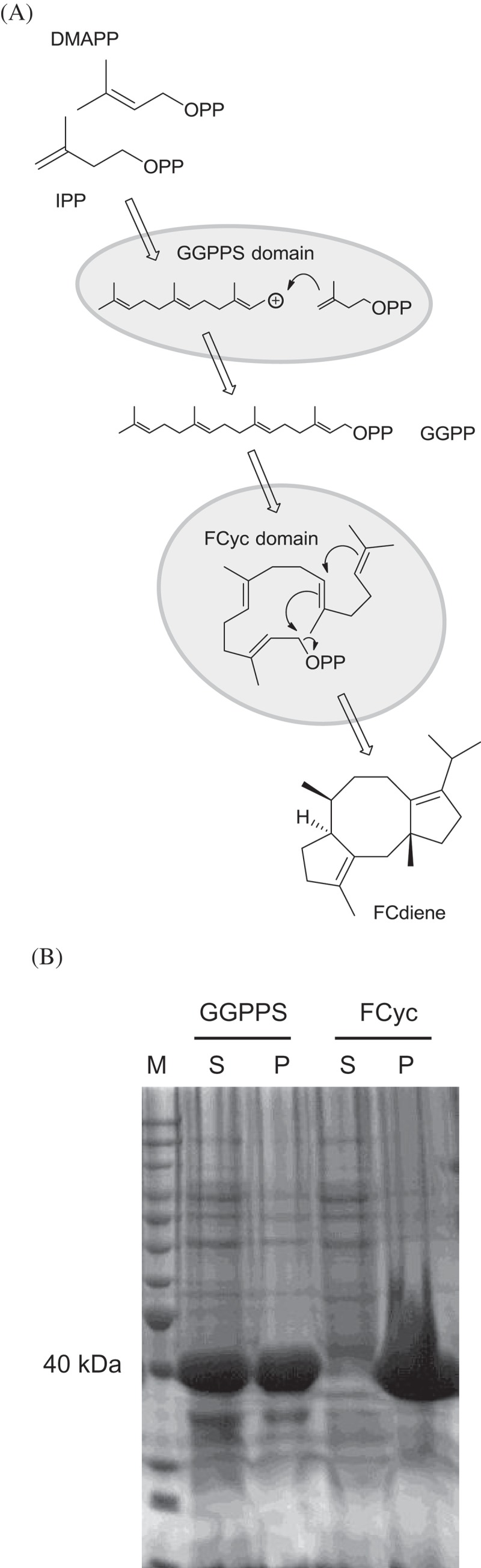
Separated expression of the two domains of the bimodular AbFS. (A) Scheme for the splitting of the AbFS. The GGPPS domain catalyses reaction from IPP and DMAPP to GGPP and the FCyc domain catalyses the cyclisation from GGPP to FCdiene. (B) SDS-PAGE showing high soluble expression of the GGPPS domain and low soluble expression of the FCyc domain.

In further experiments, we strived to identify the solubility-limiting segments of AbFS. To this end, the two catalytic domains of the enzyme (Toyomasu et al. 2007) were individually expressed in *E. coli* (Figure 6).

SDS-PAGE analysis (Laemmli 1970) revealed high soluble expression levels of the GGPPS domain. In contrast, soluble expression of the FCyc domain was low. Detection of FCdiene by GC/MS analysis in the crude extract of an expression culture of the terpene cyclase domain in *E. coli* BL21-Gold(DE3) confirmed functional activity of the stand-alone FCyc domain. Since the *directed evolution* of smaller enzymes is usually more straightforward, we conclude that the FCyc domain alone could be a better choice as starting point for *error-prone PCR* in the future.

## Summary

We have discussed different experimental approaches to improve the heterologous fermentation of terpenes in *S. cerevisiae*. Several parameters were investigated using the biosynthesis of FCdiene, the central precursor of the fusicoccane family of diterpenoids as produced by different phytopathogenic fungi, as a model system. Beginning with a metabolically engineered yeast strain, different fermentation parameters were explored. Impact of the pH during fermentation was negligible in the range of 6.5–8. Fermentation was improved under oxygen limitation and strongly limited by genetic instability of the three plasmids used to direct the fermentation. This instability was overcome by the use of a selective medium in a fed-batch mode under metabolic buffering. The formation of specific by-products was suppressed by the optimised medium. An *in situ* product removal system using a polymeric adsorbent significantly increased yield in a cost-effective way. The experiments pointed towards intrinsic limitations of the fermentation system through the diterpene cyclase–prenyl transferase fusion enzyme AbFS. Improvement of this enzyme through directed evolution was unsuccessful, but separate expression of its two catalytic domains revealed a new starting point for future enzyme engineering experiments.
